# External validation of biomarkers for immune-related adverse events after immune checkpoint inhibition

**DOI:** 10.3389/fimmu.2022.1011040

**Published:** 2022-09-29

**Authors:** Gunther Glehr, Paloma Riquelme, Jordi Yang Zhou, Laura Cordero, Hannah-Lou Schilling, Michael Kapinsky, Hans J. Schlitt, Edward K. Geissler, Ralph Burkhardt, Barbara Schmidt, Sebastian Haferkamp, James A. Hutchinson, Katharina Kronenberg

**Affiliations:** ^1^ Department of Surgery, University Hospital Regensburg, Regensburg, Germany; ^2^ Leibniz Institute for Immunotherapy, Regensburg, Germany; ^3^ Beckman Coulter Life Sciences, Krefeld, Germany; ^4^ Institute of Clinical Chemistry and Laboratory Medicine, University Hospital Regensburg, Regensburg, Germany; ^5^ Institute of Clinical Microbiology and Hygiene, University Hospital Regensburg, Regensburg, Germany; ^6^ Department of Dermatology, University Hospital Regensburg, Regensburg, Germany

**Keywords:** biomarker, checkpoint inhibition, irAEs, immune-related adverse events, validation, prediction

## Abstract

Immune checkpoint inhibitors have revolutionized treatment of advanced melanoma, but commonly cause serious immune-mediated complications. The clinical ambition of reserving more aggressive therapies for patients least likely to experience immune-related adverse events (irAE) has driven an extensive search for predictive biomarkers. Here, we externally validate the performance of 59 previously reported markers of irAE risk in a new cohort of 110 patients receiving Nivolumab (anti-PD1) and Ipilimumab (anti-CTLA-4) therapy. Alone or combined, the discriminatory value of these routine clinical parameters and flow cytometry biomarkers was poor. Unsupervised clustering of flow cytometry data returned four T cell subsets with higher discriminatory capacity for colitis than previously reported populations, but they cannot be considered as reliable classifiers. Although mechanisms predisposing some patients to particular irAEs have been described, we are presently unable to capture adequate information from pre-therapy flow cytometry and clinical data to reliably predict risk of irAE in most cases.

## Introduction

Combined checkpoint blockade with anti-PD-1 (Nivolumab) and anti-CTLA-4 (Ipilimumab) antibodies is now a standard treatment for inoperable metastatic melanoma. The clinical efficacy of dual therapy is evident from the excellent clinical response rate, progression-free survival and overall survival (1–3). However, immune-related adverse events (irAE) complicate immune checkpoint inhibitor (ICI) treatment in a high proportion of patients, which significantly impacts their quality of life (4). Although life-threatening irAEs are infrequent, even moderate reactions lead to interruption of immunotherapy, multidisciplinary management and treatment with immunosuppressive medication (5). Disruption of ICI treatment, and costs associated with monitoring or treatment of irAEs, are burdensome; therefore, reliable prognostic methods to assess an individual’s risk of irAE prior to therapy would greatly impact patient care.

Factors predisposing individual patients to irAE are incompletely understood. ICI therapy can worsen autoimmune conditions and patients with pre-existing autoimmune diseases stand a greater risk of developing other immune-mediated adverse reactions after treatment (6–8). Immunogenetics also play a role in irAE susceptibility (9–11). Prior exposure to viruses has recently surfaced as a significant predisposing factor in some patients (12). Infection with human herpesviruses (HHV) may play a particularly important role in the context of malignant melanoma. Our own studies revealed that chronic or recurrent cytomegalovirus (CMV; HHV-5) reactivation drives proliferation of virus-specific CD4^+^ effector memory T cells (T_EM_) in patients with metastatic melanoma before starting immunotherapy. These expanded T_EM_ cells are responsible for hepatitis after combined Nivolumab and Ipilimumab treatment (13–15). Similarly, others have implicated Epstein-Barr virus (EBV; HHV-4)-specific memory T cells in a case of fatal encephalitis after anti-PD-1 therapy (16). An unexpectedly high rate of seropositivity against Kaposi’s sarcoma-associated virus (KSHV; HHV-8) has been reported in Stage IV melanoma patients, again hinting at a peculiar susceptibility to HHV infections (17). Beyond ICI therapy, autoimmunity may be triggered by persistent T cell immunity against various herpesgroup viruses; for example, Hashimoto’s thyroiditis has been associated with seroconversion to roseolovirus (HHV-6) (18, 19).

Recently, many groups have reported biomarkers associated with irAE risk, which include leucocyte subsets measured in peripheral blood by flow cytometry. We systematically searched for these reports to independently assess the discriminatory value of biomarkers they identified. We found 20 relevant articles published between 2006 and 2022 that examined a range of tumor entities, treatment strategies and analytical methods (20–39). Here, we tested the general validity of these biomarkers by asking whether they predicted irAEs in a related, but non-identical clinical setting. Specifically, we asked whether any reported biomarkers measured prior to starting combined Ipilimumab and Nivolumab therapy predicted the incidence of hepatitis, colitis or thyroiditis in patients with advanced melanoma.

## Materials and methods

### Patients

This single-center, non-interventional clinical study was conducted in accordance with the Declaration of Helsinki and all applicable German and European laws and ethical standards. Blood samples were obtained from patients with Stage III/IV melanoma enrolled in an observational trial authorized by the Ethics Committee of the University of Regensburg (approval 16-101-0125) and registered with clinicaltrials.gov (NCT04158544). All participants gave full, informed written consent. The first reported case was recruited in OCT-2016 and the last reported case was recruited in JUN-2021. Patients received standard-of-care treatment according to local guidelines. Patients with unresectable metastatic disease who received first- or second-line checkpoint inhibitor therapy were initially treated with Nivolumab (αPD-1; 1 mg/kg; Bristol-Myers Squibb) plus Ipilimumab (αCTLA-4; 3 mg/kg; Bristol-Myers Squibb) for four cycles at 3 week intervals. Thereafter, patients received 3 mg/kg Nivolumab monotherapy at 3 week intervals.

### irAE grading

All irAE were assessed by an expert Dermatological Oncologist (**Supplemental Figure 1A**). ICI-related hepatitis was diagnosed when: (i) GOT, GPT, γ-GT or total bilirubin substantially deviated from pretreatment values; (ii) this change was not attributable to other causes, such as co-medication or viral disease; and (iii) liver injury was sufficiently severe that ICI therapy was suspended or stopped, or immunosuppression was given. Colitis was diagnosed according to stool frequency and consistency, abdominal discomfort, suspension or cessation of ICI therapy, and introduction of immunosuppressive treatment. Thyroiditis was diagnosed based on decreased T3/T4 and elevated TSH levels measured at routine clinic visits.

### Routine investigations

Hematological, Biochemical and Microbiological investigations were performed by accredited diagnostic laboratories at University Hospital Regensburg.

### Literature search

We searched Medline at the National Library of Medicine through the NCBI website on 11-JUN-2022. Our search terms were ‘immunotherapy’, ‘immune checkpoint inhibitor’, ‘irAEs’, ‘biomarkers’, ‘prediction’ and synonyms. We followed-up on relevant citations from articles returned in our original search. We identified 20 articles (**Supplemental Table I**) describing 59 unique biomarkers (**Supplemental Table II**).

### Flow cytometry

Step-by-step protocols for preparing and analyzing clinical samples by flow cytometry can be accessed through Nature Protocol Exchange (40). Briefly, blood was collected into EDTA-vacutainers by peripheral venepuncture before delivery to the immune monitoring lab at ambient temperature. Samples were stored at 4°C for up to 4 h until processing. Whole blood samples were stained using DURAClone reagents (DURAClone IM Phenotyping Basic Tube, B53309; DURAClone IM T Cell Subsets Tube, B53328; DURAClone IM TCRs Tube, B53340; DURAClone IM Treg Tube, B53346; DURAClone IM B Cell Tube, B53318; DURAClone IM Dendritic Cell Tube, B53351; DURAClone IM Granulocytes Tube, B88651; all from Beckman Coulter, Krefeld, Germany). For the flow cytometry anaylsis of exhausted T cells the following liquid antibodies were used (EXH_CD8 panel): CD49b FITC, 359306, BioLegend, Amsterdam, Netherlands; CD160 PE, IM3657; CD27 ECD, B26603; CD244 PC5.5, B21171; CD279 (PD-1) PC7, A78885; CD127 APC, B42026; CD8 AA700, B49181; CD3 AA750, A94680; CD4 PB, B49197 and CD45 KrO, B36294; all from Beckman Coulter, Krefeld, Germany. Data were collected with a Navios™ cytometer running Cytometry List Mode Data Acquisition and Analysis Software version 1.3 (Beckman Coulter). An experienced operator performed blinded analyses of all datasets in Kaluza version 2.1, as far as possible replicating gating strategies described in the original reports.

### Statistics

Our main dataset comprised 110 samples and 59 features extracted from publications and 9 routine clinical parameters; one missing value for GOT was imputed with the median “25” from all other 109 samples. In the extended analysis of DURAClone IM Tube panels, we extracted 80 cell population frequencies by manual gating. Additionally we included 8 clinical parameters, 9 clinical biochemistry values and 18 cell counter values in this extended feature set. One missing value of the presence of liver metastases was imputed with the median “no presence” from all other samples. Univariate analysis was performed for each condition per feature. P-values were calculated using a two-sample Wilcoxon test using a significance level of 0.05 (41). For false discovery rate (FDR) correction (42) of the p-values we used a significance level of 0.1. Discriminatory capability of the features was additionally assessed using ROC-curves and the corresponding area under the curves (AUCs). We report features with AUC > 0.65 as discriminatory. All calculations and plots were made with R 4.2.0 (43).

Models were built in leave-one-out cross-validation and the predictions for each left-out sample were gathered to report the final performance of each model. The penalized logistic regression models were built with glmnet (44) using the elastic-net (45) with an alpha=0.9, and 250 lambda steps in inner cross-validation. The random forest model was built using mlr3 (46) for each binary classification problem with alpha=0.5, num.trees=500, replace=True and splitrule=gini. We also assessed ROC-curves and the AUCs here. AUC ≃ 0 were obtained when penalization of the linear model excluded all features in multiple cross-validation steps, leading to a null-model of only the intercept.

Clustering was performed using FlowSOM (47) in CytoBank on CD45^+^ CD3^+^ T cells (DURAClone IM T Cell Subsets Tube) and CD45^+^ CD19^+^ B cells (DURAClone IM B Cell Tube). All channels were used as clustering markers except for CD3 or CD19, CD45, FSC, SSC and time. We used hierarchical consensus clustering with 10 metaclusters and 100 (T Cell Tube) or 49 (B Cell Tube) clusters. Feature standardization was applied.

## Results

### Reported biomarkers are weak predictors of irAE

Our first objective was to test the predictive performance of reported biomarkers of irAE risk in our cohort of 110 metastatic melanoma patients treated with dual checkpoint blockade. Patient characteristics are shown in **Table 1**. Reviewing the literature, we catalogued 20 publications that reported associations between irAE risk and the frequencies of 55 unique cell populations in peripheral blood or 4 routine clinical parameters (**Supplemental Table I**) (20–39). In addition, we selected another 9 routine clinical parameters with possible prognostic relevance – namely, sex, CMV seropositivity, GOT, GPT, γ-GT, total bilirubin, LDH, Protein-S100 and presence of liver metastases. Although many of these biomarkers were identified in different clinical contexts, such as anti-PD-1 monotherapy or other malignancies, we reasoned that any robust, mechanistically relevant biomarker could be reasonably expected to have some predictive capacity in closely related situations. Hence, our aim was not to directly confirm or refute any previous findings through replication, but to test whether they could be generalized.

**Table 1:** Characteristics of study cohort.

Patient cohort characteristics	
Total number of cases	110
Female	37 (33.6%)
Male	73 (66.4%)
Baseline characteristics	
Age (years)	62 (22-84)
BMI	26.6 (15.4-54.6)
Stage III	8 (7.3%)
Stage IV	102 (92.7%)
Liver metastases present	30 (27.3%)
CMV seropositive	52 (47.3%)
ANA positive	65 (59.1%)
Pretreatment	
None	3 (2.7%)
Surgical excision	102 (92.7%)
Radiosurgery	3 (2.7%)
Radiation	42 (38.2%)
Monotherapy	17 (15.5%)
IFNa therapy	9 (8.2%)
Braf/Mek inhibitor therapy	21 (19.1%)
T-VEC therapy	7 (6.4%)
Chemotherapy	6 (5.5%)
Rounds of Ipi/Nivo	
1 round	13 (11.8%)
2 rounds	24 (21.8%)
3 rounds	20 (18.2%)
4 rounds	53 (48.2%)
Complications	
Hepatitis	48 (43.6%)
Colitis	40 (36.4%)
Thyroiditis	41 (37.3%)
No complication	23 (20.9%)
1 complication	50 (45.5%)
2 complications	32 (29.1%)
3 complications	5 (4.5%)

110 patients with Stage III/IV melanoma were enrolled into the study cohort. For Age and BMI, median values were calculated. Minimum and maximum values are given in brackets. Baseline characteristics were obtained before start of Ipi/Nivo therapy.

To externally validate these 55 flow cytometry and 13 clinical features as predictors of irAEs, we performed uni- and multivariate analyses. We particularly focused on 3 common irAE – hepatitis (44%), colitis (36%) and thyroiditis (37%). Each complication was treated as an separate outcome, but we also considered the occurrence of (i) hepatitis and/or colitis, and (ii) hepatitis and/or colitis and/or thyroiditis (henceforth, “any irAE”). Hence, we tested the value of 68 features in predicting 5 clinical outcomes in our dataset of 110 cases (**Supplemental Table II**).

**Figure 1:**
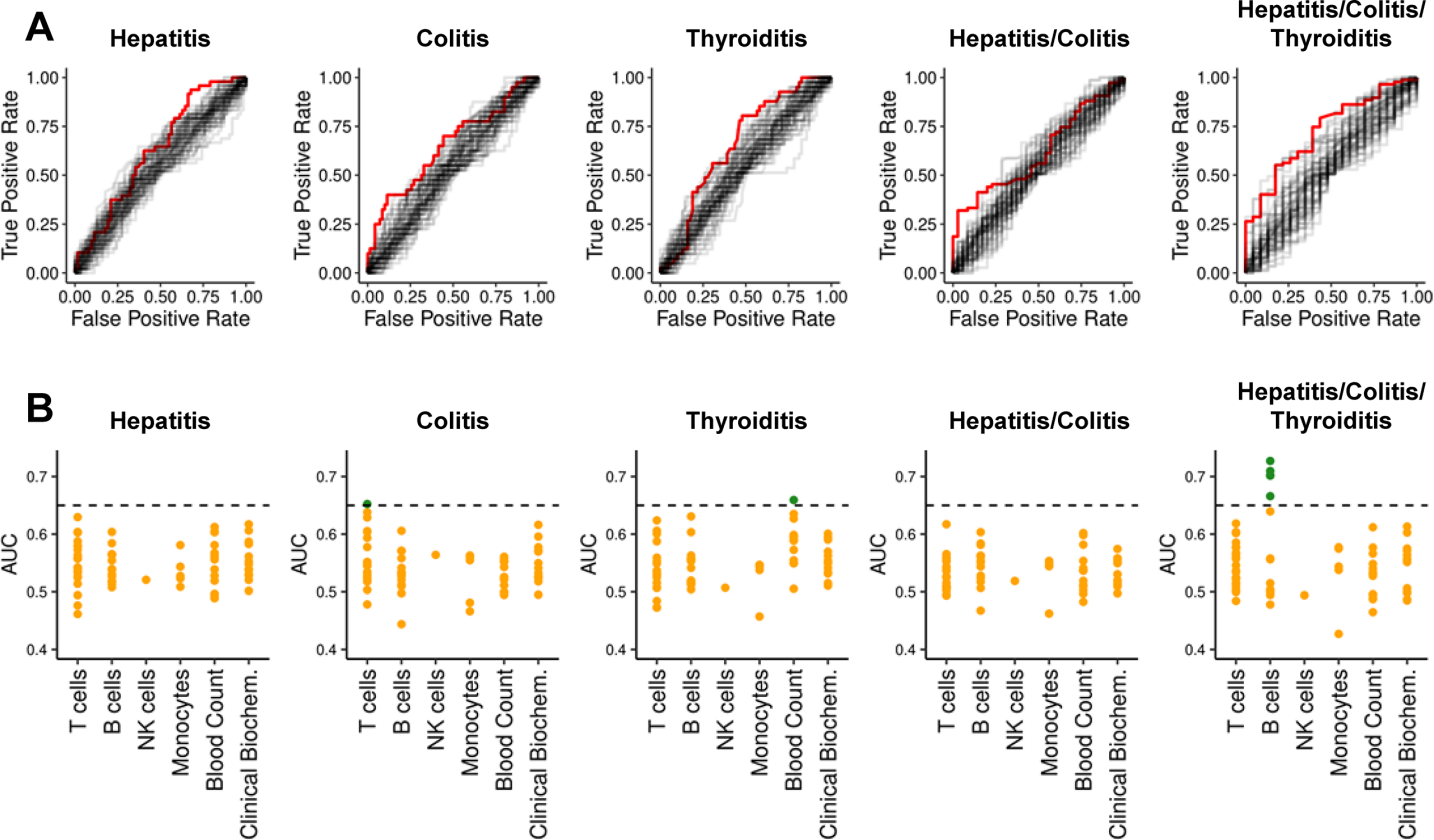
ROC-curves and AUCs for previously reported biomarkers and clinical parameters per condition. **(A)** ROC-curves for all 68 features regarding each dependent variable are shown. For each dependent variable, the features with highest AUC is highlighted in red. **(B)** AUCs from ROC-curves in subfigure **(A)** grouped according to immunological classes. The y-axis represents the AUC. Orange dots denote AUC ≤ 0.65; green dots denote AUC > 0.65.

Considering all five clinical outcomes, significant associations were discovered for 16 features using the Wilcoxon test without correcting for multiple comparison (**Supplemental Table IIIA**). However, after adjustment for multiple testing using the false discovery rate (FDR) (42), no associations with hepatitis, colitis, thyroiditis, or “hepatitis and/or colitis” were significant. Four features remained significantly associated with “any irAE” – notably, these were all B cell subsets. Next, we assessed the discriminatory capacity of all 68 features using the area under Receiver-Operating-Characteristics (ROC) curves (**Figure 1A**). An area under the curve (AUC) of 0.5 implies no discrimination, whereas a maximum AUC of 1 implies perfect discrimination. We found 7 features with AUC > 0.65 (**Supplemental Table IIIA**). Next, we asked whether these discriminatory features were capturing similar information by grouping them into immunologically relevant classes (**Figure 1B**). The most discriminatory marker for hepatitis was CD4^+^ T cell frequency (AUC=0.630) (**Supplemental Table IV**). Discriminatory markers of colitis risk related primarily to T cells, especially the frequency of CD4^+^ T cells (AUC = 0.652). The most discriminatory feature for thyroiditis risk was the platelet count (AUC = 0.659). The five most discriminatory features of “any irAE” were B cell markers (best AUC = 0.727). Unfortunately, no single biomarker was powerful enough to reliably identify predisposed individuals.

### Combining features does not improve discriminatory power

**Figure 2:**
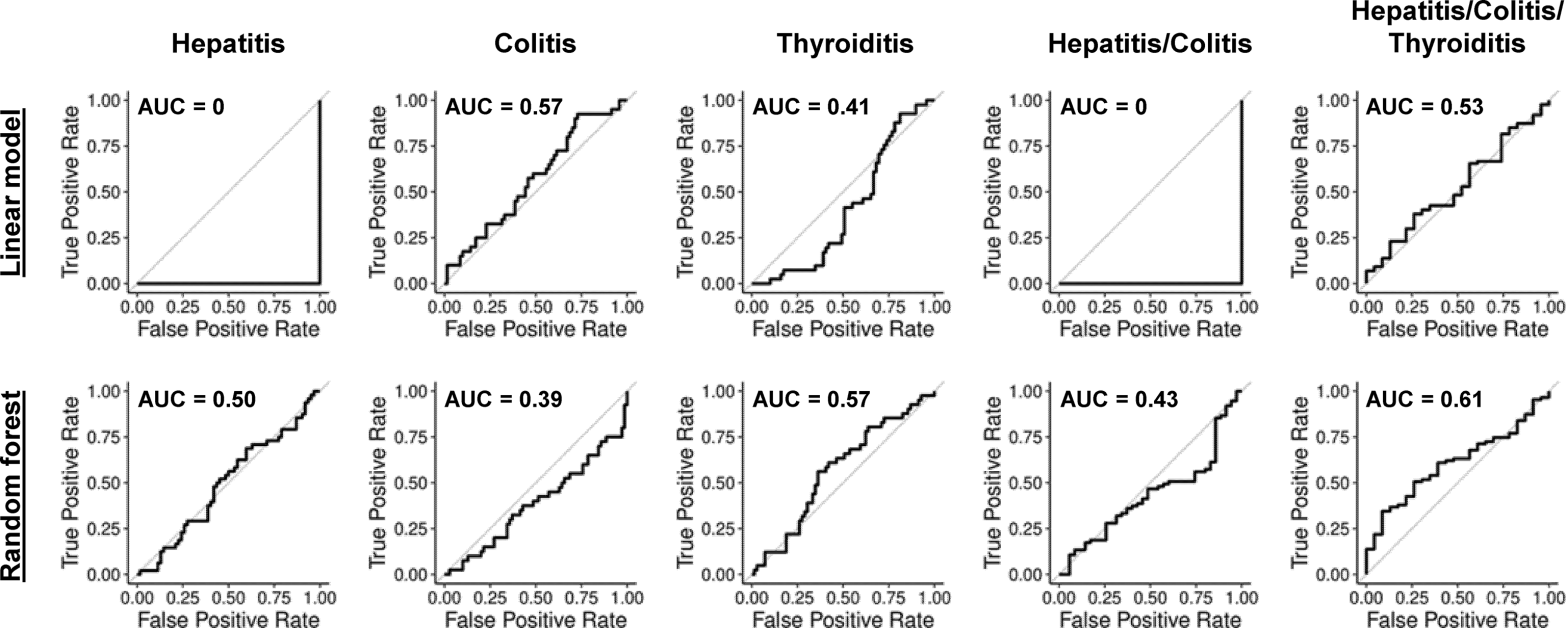
ROC-curves for linear models and random forests with previously reported biomarkers and clinical parameters. ROC-curves in LOOCV for penalized logistic regression and random forest models predicting hepatitis (AUC 0 and 0.50), colitis (AUC 0.57 and 0.39), thyroiditis (AUC 0.41 and 0.57), hepatitis and/or colitis (AUC 0 and 0.43) and hepatitis and/or colitis and/or thyroiditis (AUC 0.53 and 0.61).

We next asked whether combining previously reported features predicted irAEs better than single features alone. Therefore, we generated simple penalized logistic regression models (44) and random forest (48) analyses in leave-one-out cross-validation (LOOCV). Neither approach found reliable predictive models (**Figure 2**). AUC ≃ 0 were obtained when penalization of the linear model excluded all features in multiple cross-validation steps, leading to a null-model of only the intercept. The prediction of each left-out sample is then the mean prediction of all other samples, which always leads to incorrect class prediction.

Our inability to find a reliable predictive model combining different discriminatory biomarkers could be explained in several ways: (i) There may be no immunological predisposition to particular irAEs; (ii) immunological factors might be only partly responsible for any such predisposition; (iii) hepatitis or colitis may be the end-result of more than one immune aetiology; (iv) although we may be capturing predictive information, we might be unable to extract the signal from background noise; or (v) our selection of biomarkers might not capture all phenotypic information necessary for a reliable prediction. Importantly, the hope of finding predictive biomarkers of irAE risk that could drive personalized treatment decisions rests upon there being measurable predisposing factors of irAE. There is now good mechanistic evidence for immunological predisposition to irAEs in some cases. For instance, our group and others reported that irAE risk is predicted by oligoclonal expansion of T cells prior to immunotherapy, likely as a consequence of chronic or recurrent viral exposure (13, 20).

To investigate whether our selection of features or analytical methods were limiting the performance of our predictive models, we extended our set of biomarkers by making a finer manual re-gating of our flow cytometry data before repeating our uni- and multivariate analyses. After correction for multiple testing, no extended-set features were significantly associated with hepatitis, colitis, thyroiditis or “hepatitis and/or colitis” risk. However, three B cell subpopulations were significant markers of “any irAE” (**Supplemental Table IIIB**). The extended-set feature that returned the highest AUCs in prediction of hepatitis was CD4^+^ T_EM_ (AUC = 0.677), whereas colitis was weakly predicted by immature neutrophils (AUC = 0.670) (**Supplemental Table IV**). Unfortunately, combining the extended-set features did not return a more stable predictive model for any of the outcomes (**Supplemental Figure 1B**).

### ICI-related hepatitis may have more than one cause

We previously reported that CD4^+^ T_EM_ expansion in CMV-seropositive patients before therapy is a strong predictor of hepatitis risk after combined Nivolumab and Ipilimumab treatment (13). We were able to rederive this result in a subset of patients comprising the validation set from our original publication (n=45) plus an additional 30 patients added in this study: The AUC for CD4^+^ T_EM_ (%) was 0.729. In addition, we used the full dataset to discover another 12 markers of CMV IgG^+^ hepatitis with AUC > 0.65, which were mainly T cell subsets (**Supplemental Table IIIC**). Interestingly, for the CMV IgG^-^ samples, monocyte frequency (AUC = 0.705) and absolute numbers (AUC = 0.657) predicted hepatitis risk, suggesting there may be more than one aetiological route to ICI-related liver inflammation (**Supplemental Table IIID** & **Supplemental Table IV**).

### Unsupervised clustering returns new predictive features

It is conceivable our flow cytometry dataset captured predictive information about irAE risk, but that our manual gating strategy failed to identify the most informative cell subsets. Therefore, we applied an unsupervised clustering algorithm (FlowSOM) to samples stained with B cell or T cell markers, then used clusterwise cell abundances as predictive features (47). Univariate analyses after clustering of B cell markers identified no new features with greater discriminatory value than previously considered features (**Supplemental Tables IIIE, F**). Furthermore, the top-performing models after combining B cell (meta-)clusters in LOOCV were not superior to single features alone (**Supplemental Table IV**, **Supplemental Figures 2A, B**).

Likewise, clustering T cells revealed no better discriminatory features for hepatitis, thyroiditis, “hepatitis or colitis” or “any irAE” (**Supplemental Tables IIIG, H**, **Supplemental Table IV** & **Supplemental Figure 2C**). Surprisingly, 4 clusters (C45, C50, C56 and C63) were significantly associated with colitis after FDR correction. These clusters returned AUCs of 0.690, 0.709, 0.711 and 0.713, respectively – hence, they showed greater discriminatory value than previously considered features (**Supplemental Table IIIH**). Unfortunately, combining C45, C50, C56 and C63 in LOOCV did not improve their predictive performance (**Supplemental Figure 2D**).

**Figure 3:**
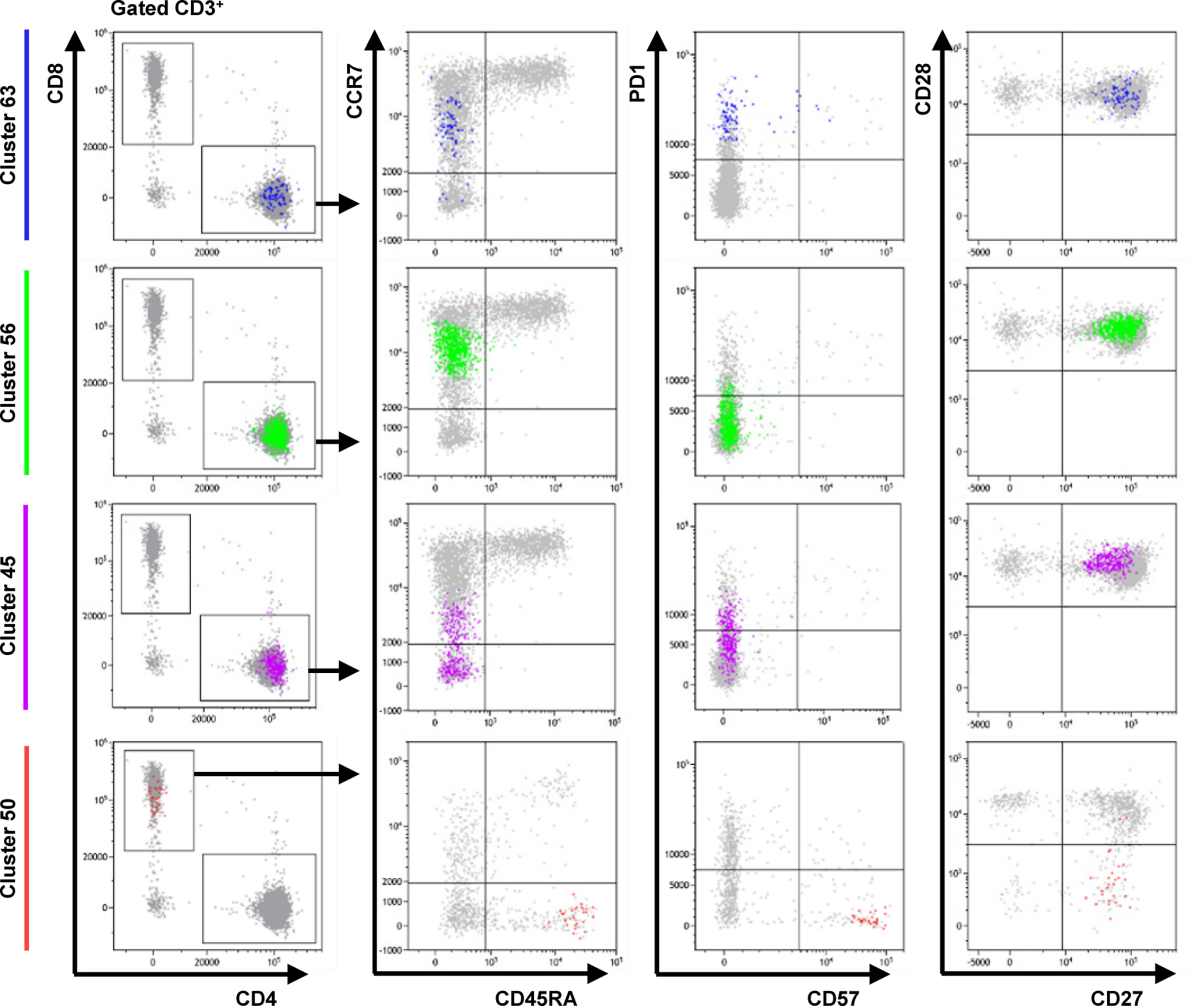
Phenotype of cells in FlowSOM clusters associated with colitis. Dot plots show the phenotype of the cells in each cluster (color) and all gated cells for reference (grey). Clusters 63 and 56 are CD4^+^ CD45RA^-^ CCR7^int^ CD27^+^ CD28^+^ CD57^-^ T cells that differ only in expression of PD-1. Cluster 45 is CD4^+^ CD45RA^-^ CCR7^low/-^ PD-1^int^ CD27^+^ CD28^+^ CD57^-^ T cell population. Cluster 50 represents a CD8^+^ CD45RA^+^ CCR7^-^ CD27^+^ CD28^-^ PD-1^-^ CD57^+^ T_EMRA_ subpopulation. Data from one representative patient.

We next asked why these particular T cell clusters might encode more information about colitis risk than other T cell subsets. C63, C56 and C45 were CD4^+^ memory T cells with a CD45RA^-^ CCR7^int/-^ CD27^+^ CD28^+^ CD57^-^ phenotype, possibly representing transitional states between recently activated central memory (T_CM_) and T_EM_ cells (**Figure 3**). Apart from CCR7 expression, these T cell clusters differed only in PD-1 expression. C50 was a minor population of CD8^+^ CD45RA^+^ CCR7^-^ CD27^+^ CD28^-^ PD-1^-^ CD57^+^ T_EMRA_ cells. We speculate that C50 overlaps with a non-exhausted, recirculating subset of CD8^+^ T_EMRA_ cells that others have reported as important for maintaining anti-viral immunity (49). Our results suggest that patients at risk of ICI-related colitis might have on-going immune responses – possibly against subclinical viral infections – and that our predictive features actually capture information about the rate of T_CM_ to T_EM_ differentiation.

## Discussion

Reproducibility studies using external data are an important validation step in clinical biomarker development. Here, we showed that previously reported flow cytometry-based biomarkers of irAE are not generally reliable enough to predict hepatitis, colitis or thyroiditis as a basis for clinical decision-making. Promisingly, however, unsupervised clustering revealed four T cell subpopulations associated with risk of colitis that returned AUC > 0.65, which we take as a sign that better predictions of irAE risk might be possible with a refined marker selection and more sophisticated computational methods. We conclude that deeper phenotyping of monocytes and CD4^+^ memory T cells transitioning between T_EM_ and T_CM_ might lead to more informative biomarkers in future.

## Data availability statement

The original contributions presented in the study are included in the article/**Supplementary Material**. Further inquiries can be directed to the corresponding author.

## Ethics statement

The studies involving human participants were reviewed and approved by Ethics Committee of the University of Regensburg. The patients/participants provided their written informed consent to participate in this study.

## Author contributions

GG performed statistical evaluation and wrote the manuscript. JY performed literature search. PR and LC analyzed data and revised the manuscript. H-LS revised the manuscript. MK provided expert flow cytometry advice. HS, EG and RB provided infrastructural support and critical feedback. BS provided expert virological opinion. SH provided clinical samples and expert dermatological opinion. JH designed study and wrote the manuscript. KK performed experiments, analyzed data and wrote the manuscript. All authors contributed to the article and approved the submitted version.

## Funding

This work was supported by Bristol-Myers Squibb (BMS) Immune Oncology Foundation (Award FA19-009). LC is a Marie Skłodowska-Curie Research Fellow affiliated with INsTRuCT and receives funding from the European Union’s Horizon 2020 research and innovation programme (Award 860003).

## Acknowledgments

The authors are grateful to The Bristol Myers Squibb Foundation for Immuno-Oncology for funding this work. We are very grateful for Beckman Coulter Life Sciences’ continuing support of our research. This work would not have been possible without the outstanding technical support of Erika Ostermeier and Joachim Schweimer.

## Conflict of interest

MK is a Beckman Coulter Life Sciences associate. SH has received consulting fees and speaker’s honoraria from BMS and Merck Sharp & Dohme (MSD).

The remaining authors declare that the research was conducted in the absence of any commercial or financial relationships that could be construed as a potential conflict of interest.

## Publisher’s note

All claims expressed in this article are solely those of the authors and do not necessarily represent those of their affiliated organizations, or those of the publisher, the editors and the reviewers. Any product that may be evaluated in this article, or claim that may be made by its manufacturer, is not guaranteed or endorsed by the publisher.

## Abbreviations

AUC, Area-under-the-curve; BMI, body mass index; CMV, cytomegalovirus; EBV, Epstein-Barr virus; FDR, false discovery rate; γ-GT, gamma-glutamyltransferase; GOT, glutamic oxaloacetic transaminase; GPT, glutamic pyruvic transaminase; HHV, human herpesvirus; ICI, Immune Checkpoint Inhibitor; irAE, immune-related adverse event; KSHV, Kaposi’s sarcoma-associated virus; LDH, lactate dehydrogenase; LOOCV, leave-one-out cross-validation; MLR, monocyte-to-lymphocyte ratio; ROC, receiver operating characteristic.
