# Supplementary material for: External validation of biomarkers for immune-related adverse events after immune checkpoint inhibition
Source: Front Immunol. 2022 Sep 29;13:1011040. doi: 10.3389/fimmu.2022.1011040 (PMC9556693; doi:10.3389/fimmu.2022.1011040)
Supplement: Supplementary file 2 [file DataSheet_1.pdf]

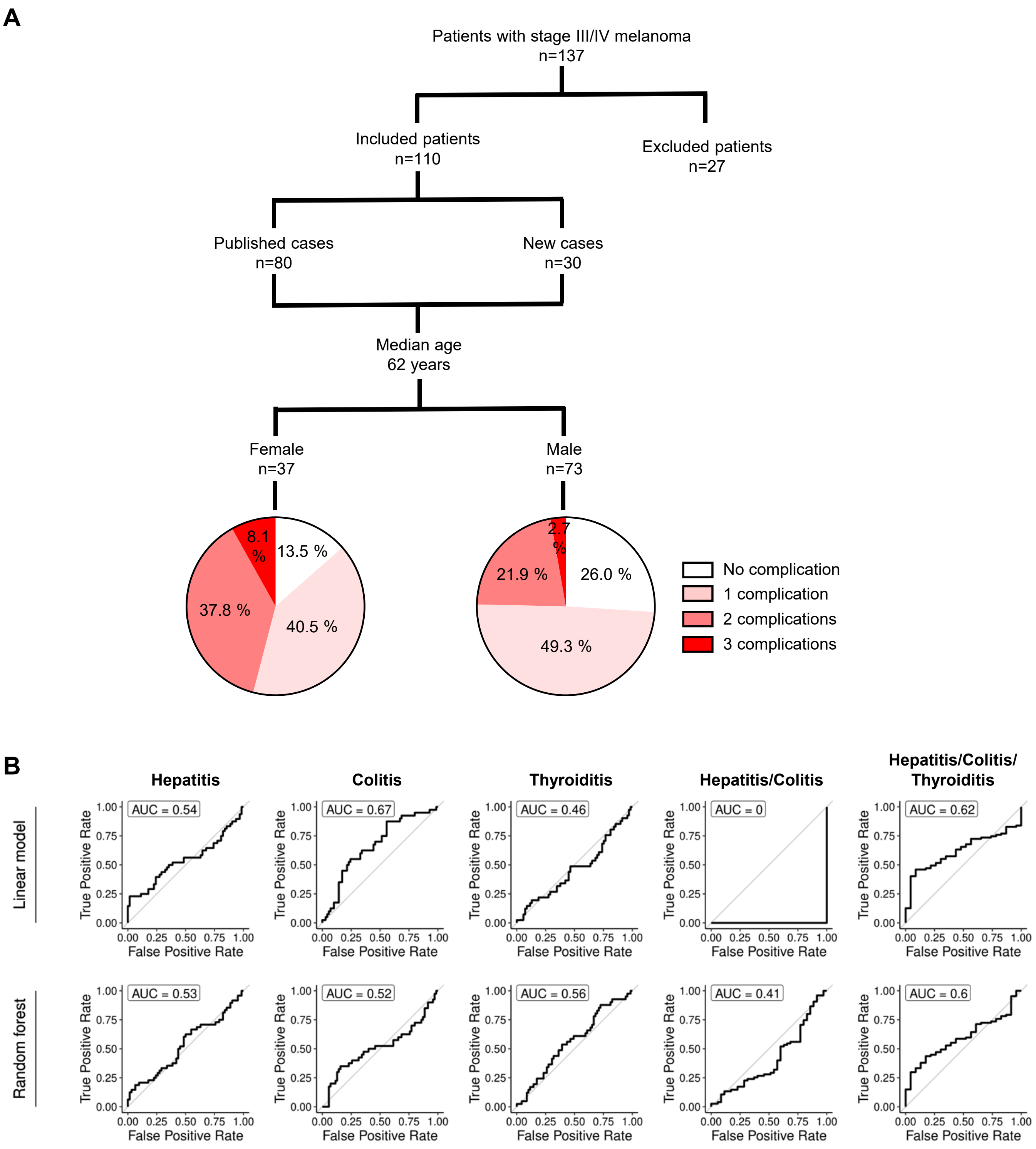

Supplemental Figure 1. **(A)** *Stage III/IV melanoma patients mainly developed one or more complications after treatment with Ipilimumab and Nivolumab dual checkpoint blockade therapy.* EDTA blood samples of 137 patients with stage III/IV melanoma were collected from OCT-2016 until JUN-2021. Because of missing data or lost to follow up 27 patients had to be excluded from the analysis. In the remaining cohort with 110 patients, data from 80 patients have been published previously [Hutchinson et al. (2021) *Nat Commun* 12, 1439, https://doi.org/10.1038/s41467-021-21572-y] whereas 30 cases were newly recruited. The median age of the patient cohort was 62 years with 37 female and 73 male patients. Most of the patients developed one or more complications (hepatitis, colitis or thyroidits) after treatment with Ipilimumab and Nivolumab. **(B)** *ROC-curves for linear models and random forests using standardized gates from DURAClone IM Tube panels as features.* ROC-curves in LOOCV for penalized logistic regression and random forest model. Features were extracted from standardized DURAClone IM Tube panels. We modelled hepatitis (AUC 0.54 and 0.53), colitis (AUC 0.67 and 0.52), thyroiditis (AUC 0.46 and 0.56), hepatitis and/or colitis (AUC 0 and 0.41) and hepatitis and/or colitis and/or thyroiditis (AUC 0.62 and 0.6).

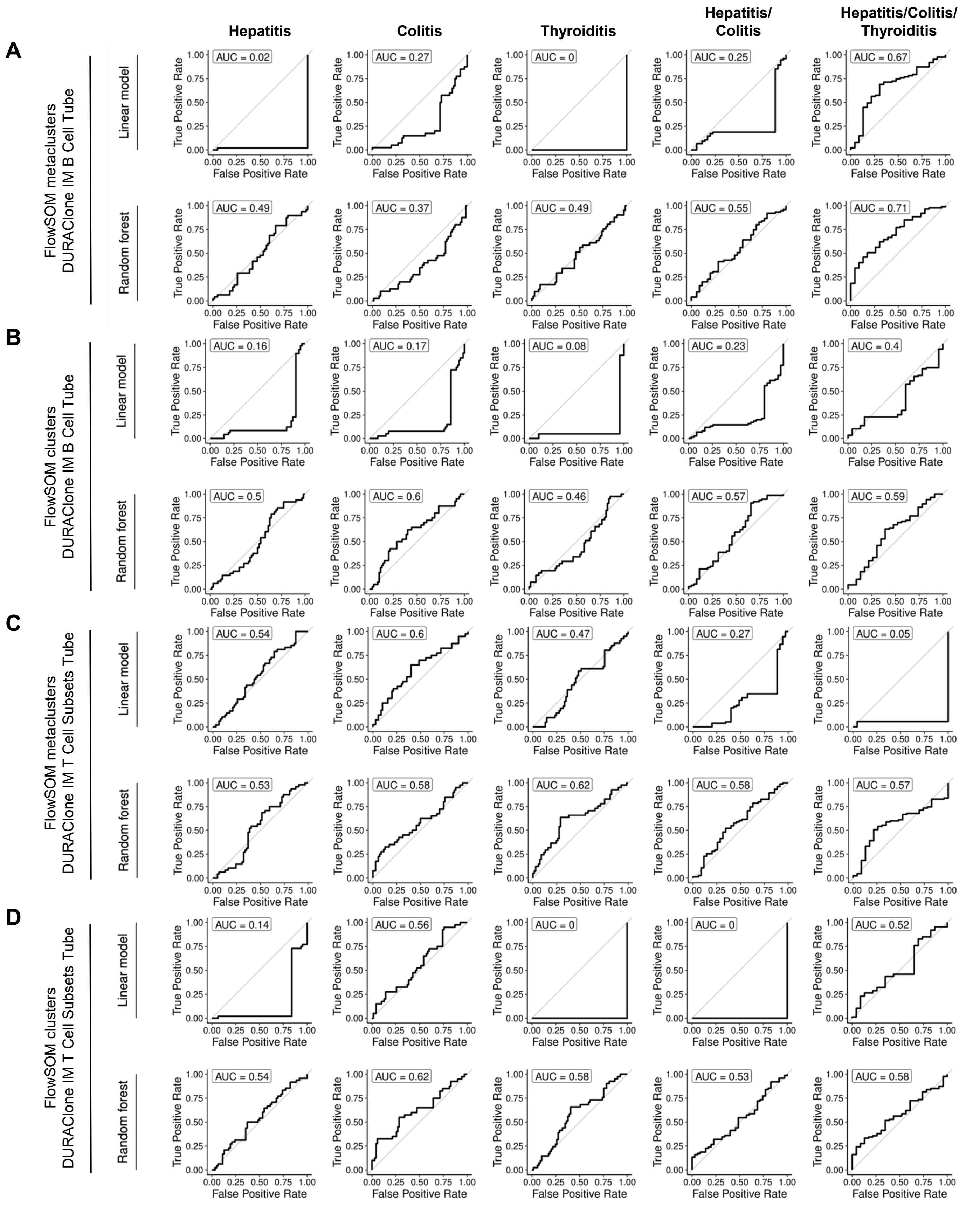

Supplemental Figure 2. *ROC-curves for linear models and random forests using FlowSOM metaclusters and clusters from DURAClone IM Tubes.* ROC-curves in LOOCV for penalized logistic regression and random forest models using FlowSOM metaclusters and clusters from DURAClone IM Tubes as features. We modelled hepatitis, colitis, thyroiditis, hepatitis and/or colitis and hepatitis and/or colitis and/or thyroiditis. **(A)** FlowSOM metaclusters from DURAClone IM B Cell Tube. **(B)** FlowSOM clusters from DURAClone IM B Cell Tube. **(C)** FlowSOM metaclusters from DURAClone IM T Cell Subsets Tube. **(D)** FlowSOM clusters from DURAClone IM T Cell Subsets Tube. **(A-D)** The combined models did not lead to an improved AUC in comparison to the previously reported biomarkers.
